# Case Report: Phosphaturic mesenchymal tumor arising from the jugular bulb without symptomatic osteomalacia

**DOI:** 10.3389/fsurg.2026.1994124

**Published:** 2026-10-14

**Authors:** Sen Yang, Jianan He, Hui Yang, Bin Hu, Ziyu Chen, Zhonghui Wen

**Affiliations:** 1 Department of Neurosurgery, The First Affiliated Hospital of Kunming Medical University, Kunming, Yunnan, China; 2 Second Department of Surgery, Menglian County People's Hospital, Pu’er, Yunnan, China; 3 Department of Nephrology, Pu’er People's Hospital, Pu’er, Yunnan, China; 4 Department of Neurosurgery, Pu’er People's Hospital, Pu’er, Yunnan, China

**Keywords:** hypophosphatemia, infratemporal fossa approach, jugular bulb, jugular foramen, phosphaturic mesenchymal tumor, skull base surgery, tumor-induced osteomalacia

## Abstract

**Background:**

Phosphaturic mesenchymal tumor (PMT) is a rare mesenchymal neoplasm that may be associated with tumor-induced osteomalacia (TIO), typically through fibroblast growth factor 23 (FGF23)-mediated phosphate wasting. Skull-base PMTs are uncommon, and lesions centered on the jugular foramen or jugular bulb are particularly rare.

**Case presentation:**

A 32-year-old man presented with progressive headache and dizziness for 2 months without bone pain, muscle weakness, or pathological fractures. Routine preoperative biochemical testing showed mild hypophosphatemia (0.78 mmol/L); this finding prompted further evaluation with a 24-h urinary phosphate measurement, which was elevated (48 mmol/24 h), together with mildly elevated alkaline phosphatase (147 U/L). Contrast-enhanced magnetic resonance imaging demonstrated a 2.4 × 2.5 × 2.0 cm enhancing mass in the left jugular foramen region. Preoperative CT showed a mixed-density lesion with multiple patchy calcifications, and bone-window CT demonstrated adjacent skull-base bone destruction involving the left jugular foramen and left hypoglossal canal. Magnetic resonance venography showed absent flow signals in the ipsilateral transverse sinus, sigmoid sinus, and internal jugular vein. The tumor was resected through a Fisch type A infratemporal fossa approach. Histopathological and immunohistochemical findings supported the diagnosis of PMT. The patient had no new postoperative neurological deficit or clinically apparent hearing deterioration. Without phosphate or vitamin D supplementation, serum phosphate, urinary phosphate, and alkaline phosphatase normalized after resection and remained within the reference range at 3 months.

**Conclusion:**

PMT should be considered in the differential diagnosis of unusual jugular foramen masses, particularly when abnormalities of phosphate metabolism are present. This case illustrates that a jugular bulb PMT may be identified before symptomatic osteomalacia develops. In this patient, gross-total resection was followed by normalization of phosphate-related biochemical abnormalities and a favorable short-term clinical outcome.

## 1 Introduction

Phosphaturic mesenchymal tumor (PMT) is a rare mesenchymal neoplasm, usually benign, that is the most common tumor associated with tumor-induced osteomalacia (TIO). Many clinically functional PMTs secrete excess fibroblast growth factor 23 (FGF23), which decreases renal tubular phosphate reabsorption and suppresses 1*α*-hydroxylase activity, resulting in renal phosphate wasting, reduced 1,25-dihydroxyvitamin D, hypophosphatemia, and osteomalacia (1, 2). Patients with established TIO commonly present with progressive bone pain, muscle weakness, gait impairment, and pathological fractures, and the diagnosis may be delayed because these manifestations are nonspecific (1, 2).

PMTs can arise throughout the body and most often occur in the extremities. Craniofacial and intracranial lesions are much less common, and skull-base tumors are reported mainly as isolated cases or small series (3, 4). Among intracranial PMTs, the jugular foramen is an exceptionally uncommon site (3). The complex anatomy of this region and the nonspecific radiological appearance of PMT can make preoperative diagnosis and surgical management difficult.

We report a pathologically confirmed PMT arising within the left jugular bulb and associated with occlusion of the ipsilateral sigmoid sinus and internal jugular vein. The case is notable for mild abnormalities of phosphate metabolism without symptomatic osteomalacia and for gross-total resection through a Fisch type A infratemporal fossa approach.

## 2 Case presentation

### 2.1 Patient information and clinical findings

A 32-year-old man presented with a 2-month history of progressively worsening headache and dizziness. The headache was aggravated by physical activity and improved with rest. During the preceding weeks, he also reported reduced appetite and poor sleep, but denied fever or significant weight change. He had no history of bone pain, muscle weakness, or pathological fracture. Neurological examination revealed no focal deficit. Clinical hearing examination was unremarkable, and fundoscopic examination was normal without evidence of papilledema. Cranial computed tomography (CT) performed at a local hospital identified an intracranial mass, and the patient was referred to our center for further evaluation.

Serum phosphate, which is included in routine preoperative biochemical testing at our institution, was mildly reduced at 0.78 mmol/L (reference range, 0.85–1.51 mmol/L). This abnormal result prompted further evaluation with a 24-h urinary phosphate measurement, which was elevated at 48 mmol/24 h (reference range, 13–42 mmol/24 h). Alkaline phosphatase (ALP) was mildly elevated at 147 U/L (reference range, 45–125 U/L), whereas serum calcium, serum creatinine, and urinary creatinine were within their respective reference ranges. Serum FGF23, parathyroid hormone, 25-hydroxyvitamin D, and 1,25-dihydroxyvitamin D were not measured. The patient did not receive phosphate or vitamin D supplementation during the perioperative period.

### 2.2 Diagnostic assessment

Contrast-enhanced MRI revealed a mass in the left jugular foramen region measuring approximately 2.4 × 2.5 × 2.0 cm. Preoperative CT demonstrated a mixed-density lesion with multiple patchy calcifications. Bone-window CT showed adjacent skull-base bone destruction involving the left jugular foramen and the left hypoglossal canal (Figure 1D). Magnetic resonance venography showed absent flow signals in the left transverse sinus, sigmoid sinus, and internal jugular vein (Figure 1E). MR perfusion was not performed. The imaging findings were not specific enough to establish a definitive preoperative pathological diagnosis, and preoperative tumor embolization was not planned.

**Figure 1:**
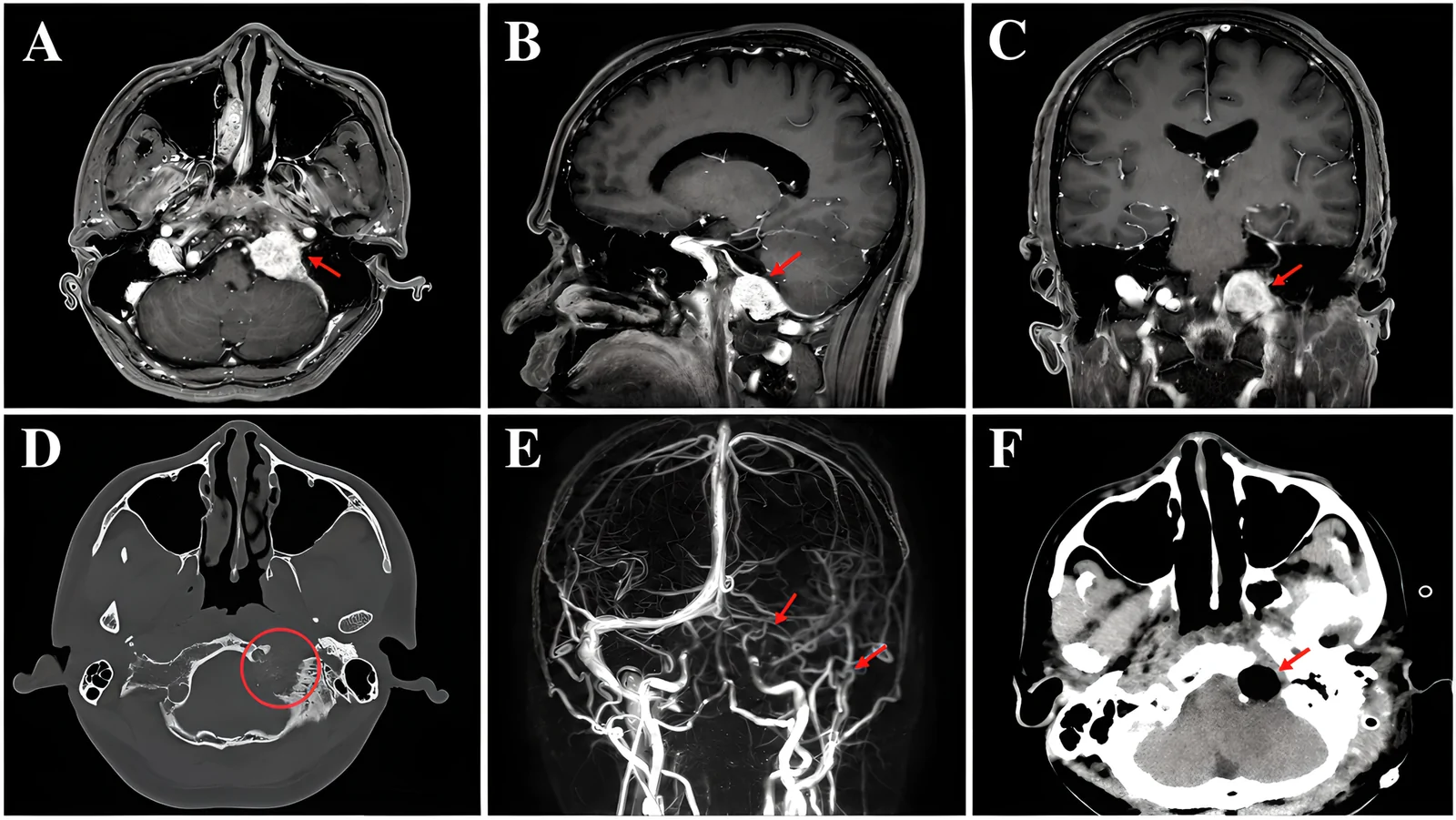
Preoperative and postoperative imaging of the left jugular foramen tumor. **(A–C)** Contrast-enhanced MRI (axial, sagittal, and coronal) showing an enhancing mass in the left jugular foramen region (red arrows), measuring approximately 2.4 × 2.5 × 2.0 cm. **(D)** Preoperative CT showing a mixed-density lesion with multiple patchy calcifications. Bone-window CT demonstrates adjacent skull-base bone destruction involving the left jugular foramen and the left hypoglossal canal (red circle). **(E)** Preoperative MRV demonstrating absence of flow signal in the left transverse sinus, sigmoid sinus, and internal jugular vein (red arrows). **(F)** Postoperative CT scan confirming gross-total resection of the tumor (red arrow).

### 2.3 Timeline

### 2.4 Therapeutic intervention

After multidisciplinary evaluation, surgical resection was undertaken using a Fisch type A infratemporal fossa approach (Figure 2A). The transverse–sigmoid sinus junction, sigmoid sinus, and jugular bulb were exposed after mastoid drilling, and the mastoid segment of the facial nerve was skeletonized without facial nerve transposition. The cervical internal jugular vein was exposed. After opening the lateral wall of the sigmoid sinus and jugular bulb, a soft, highly vascular tumor was identified within the jugular bulb (Figures 2B,C). Temporary control of the external carotid and occipital arteries was used to facilitate hemostasis. The tumor was carefully dissected and removed in a piecemeal fashion to achieve gross-total resection. Bleeding from the inferior petrosal sinus after tumor removal was controlled with gelatin-based hemostatic material, and the mastoid defect was reconstructed with artificial bone. Postoperative CT confirmed gross-total removal (Figure 1F).

**Figure 2:**
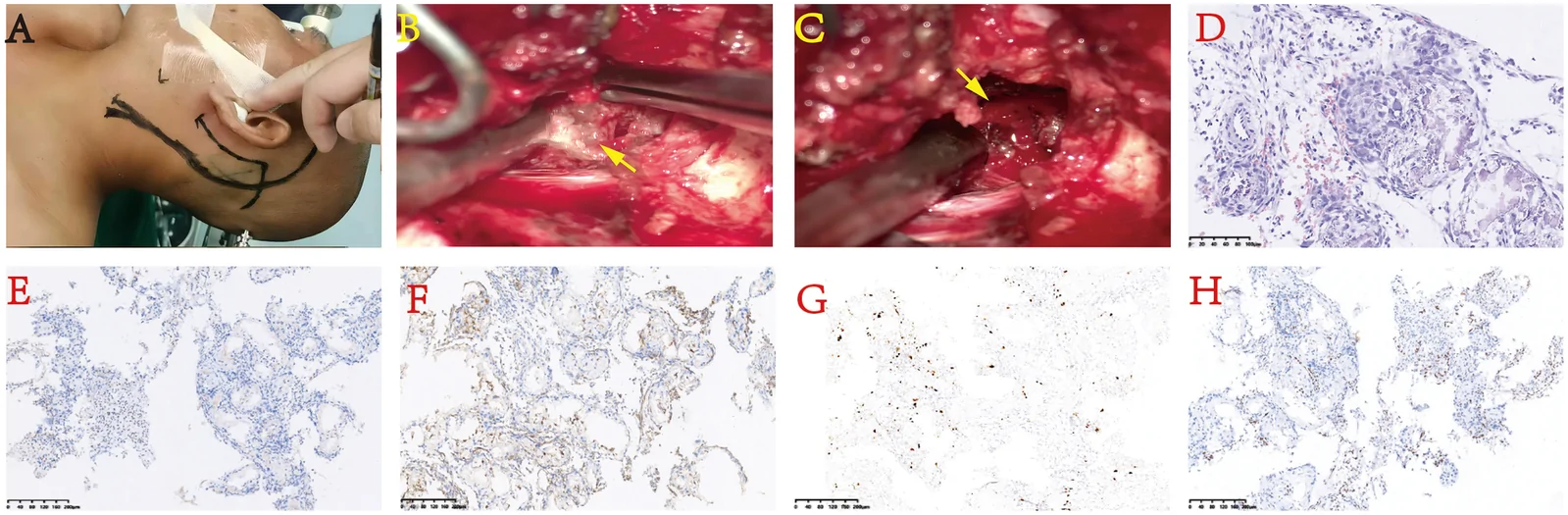
Surgical approach, intraoperative findings, and pathological examination. **(A)** Skin incision for the Fisch type A infratemporal fossa approach. **(B,C)** Intraoperative exposure of the jugular bulb tumor (yellow arrows). **(D)** Hematoxylin and eosin staining. **(E)** ERG immunostaining. **(F)** SSTR2A immunostaining. **(G)** SATB2 immunostaining. **(H)** Ki-67 immunostaining.

### 2.5 Pathological findings

Gross examination showed multiple gray-white tissue fragments measuring approximately 1.5 × 1.0 × 0.8 cm in aggregate. Histopathological examination supported a diagnosis of PMT (Figures 2D–H). Immunohistochemistry showed focal positivity for CD56, ERG, and SATB2, partial positivity for SSTR2A and S-100, and a Ki-67 labeling index of approximately 2%. EMA, PR, SOX10, GFAP, brachyury, p63, synaptophysin, CD68, chromogranin A, cadherin, and D2-40 were negative.

### 2.6 Follow-up, outcomes, and patient perspective

The postoperative course was uneventful, with no new neurological deficit. Headache and dizziness substantially improved. No obvious change in clinical hearing was noted compared with the preoperative status. Biochemical abnormalities normalized by postoperative day 7 and remained within the reference range at 1 and 3 months (Table 1). No phosphate or vitamin D supplementation was administered during this period. At the 3-month follow-up, the patient had returned to normal daily activities without new neurological symptoms and reported satisfaction with the treatment outcome. He expressed willingness to share his clinical experience to improve awareness of this rare disease.

**Table 1:** Timeline of the clinical course and biochemical changes.

Time point	Clinical event	Serum phosphate	24-h urinary phosphate	ALP	Outcome
2 months before admission	Progressive headache and dizziness	—	—	—	Symptoms progressed
Admission	Jugular foramen mass identified	0.78 mmol/L	48 mmol/24 h	147 U/L	Mild phosphate-metabolism abnormality
Surgery	Gross-total resection via Fisch type A infratemporal fossa approach	—	—	—	PMT confirmed histologically
Postoperative day 7	Biochemical follow-up	1.23 mmol/L	26 mmol/24 h	90 U/L	Biochemical normalization
1 month	Clinical and biochemical follow-up	1.08 mmol/L	31 mmol/24 h	105 U/L	Stable; no new neurological deficit
3 months	Clinical and biochemical follow-up	1.29 mmol/L	25 mmol/24 h	92 U/L	Normal daily activities; no new neurological deficit

## 3 Discussion

PMT can produce two broad categories of clinical manifestations: systemic manifestations related to disturbances of phosphate metabolism and local symptoms caused by the anatomical effects of the tumor (1–5, 9, 10). The present patient was unusual because the lesion was detected before the development of symptomatic osteomalacia. Although he denied bone pain, muscle weakness, and pathological fractures, laboratory testing showed mild hypophosphatemia, increased 24-h urinary phosphate excretion, and mildly elevated ALP. Because serum FGF23 and a complete phosphate-metabolism profile were not available, these findings cannot establish FGF23-mediated TIO. Nevertheless, the postoperative normalization of these biochemical abnormalities in the absence of phosphate or vitamin D supplementation supports a temporal association with tumor removal.

Diagnosis of PMT requires integration of biochemical, radiological, and pathological findings. In typical TIO, hypophosphatemia and renal phosphate wasting are often accompanied by elevated or inappropriately normal FGF23 and reduced 1,25-dihydroxyvitamin D (1, 2, 9, 10, 12). In the present case, the 24-h urinary phosphate value suggested abnormal phosphate handling, but a definitive assessment of renal tubular phosphate wasting could not be made because paired measurements required to calculate fractional tubular phosphate reabsorption or TmP/GFR were not available. Functional somatostatin-receptor imaging, particularly 68Ga-DOTATATE PET/CT, is valuable for localizing occult or residual PMTs, whereas CT and MRI are important for anatomical characterization and surgical planning (5, 10, 11). Radiologically, jugular foramen PMTs may overlap with more common lesions such as paraganglioma, schwannoma, and meningioma. In this patient, CT additionally demonstrated multiple patchy calcifications and adjacent skull-base bone destruction involving the jugular foramen and hypoglossal canal. The definitive diagnosis therefore relied on histopathological and immunohistochemical examination. Recurrent molecular alterations described in PMT include FN1-FGFR1 and, less commonly, FN1-FGF1 fusions (12–14).

Intracranial PMTs are exceptionally uncommon. Recent systematic reviews have emphasized the rarity and heterogeneous anatomical distribution of intracranial disease (6, 7). The literature also contains lateral skull-base PMTs arising from or extending to the temporal bone, jugular foramen, basiocciput, and adjacent structures (15–18). Table 2 summarizes representative cases most relevant to the present lesion. Importantly, some previously reported tumors originated in adjacent skull-base bone and secondarily involved the jugular foramen. In the present case, the intraoperative finding of a soft, hypervascular tumor located within the jugular bulb supports a primary jugular bulb/jugular foramen lesion.

**Table 2:** Representative reported phosphaturic mesenchymal tumors of the jugular foramen and adjacent lateral skull base.

Study	Age/Sex	Site	Status	Treatment	Reported outcome	Ref.
Gunawat et al., 2019	53/F	Left jugular foramen/posterior skull base	Initial	Preoperative embolization + surgery	NED at 6 months	(15)
Kaylie et al., 2006	46/F	Temporal bone/lateral skull base	Recurrent	Repeat surgery	Recurrence after prior resections	(16)
Argersinger et al., 2022	62/M	Right jugular foramen	Initial	Surgery	Complete resection reported	(17)
Basu and Fargose, 2016	53/F	Left basiocciput with jugular foramen involvement	Recurrent/inoperable	177Lu-DOTATATE PRRT after prior surgeries	Symptomatic and imaging improvement	(18)
Present case	32/M	Left jugular bulb/jugular foramen	Initial	Gross-total resection	Biochemical normalization; no new neurological deficit at 3 months	—

Complete surgical resection remains the preferred treatment for resectable PMT and is often followed by improvement in phosphate metabolism (2, 9, 19). Jugular foramen surgery is technically demanding because of the close relationship of the tumor to the lower cranial nerves, facial nerve, internal carotid artery, internal jugular vein, and major dural venous sinuses (8). Surgical approach selection should be individualized according to tumor location, extension, hearing status, and its relationship to critical neurovascular structures. Depending on these features, alternative approaches may include retrosigmoid, far-lateral, transmastoid/infralabyrinthine, or combined approaches. In the present case, the Fisch type A infratemporal fossa approach provided direct access to the jugular bulb and allowed gross-total removal. The mastoid facial nerve was skeletonized but was not transposed. Careful vascular control was important because of the marked tumor vascularity. For unresectable or recurrent somatostatin-receptor-positive disease, peptide receptor radionuclide therapy has been reported as a potential treatment option (18).

This case has several limitations. First, serum FGF23, PTH, 25-hydroxyvitamin D, and 1,25-dihydroxyvitamin D were not measured, and no stored preoperative serum or plasma sample was available for retrospective FGF23 testing. In addition, paired biochemical measurements required for calculation of fractional tubular phosphate reabsorption or TmP/GFR were not available; therefore, the elevated 24-h urinary phosphate value should be interpreted cautiously. Second, standardized pre- and postoperative audiological testing was not performed, although clinical hearing examination was unremarkable preoperatively and no obvious postoperative hearing deterioration was observed. Third, postoperative 68Ga-DOTATATE PET/CT was not performed because this examination is not available at our institution. Finally, follow-up is currently limited to 3 months, and longer clinical and radiological surveillance is required to assess durable local control and recurrence.

The principal clinical lesson is that a PMT may present as a skull-base mass with only mild abnormalities of phosphate metabolism before symptomatic osteomalacia becomes clinically apparent. Mild hypophosphatemia should therefore not be overlooked in patients with unusual jugular foramen tumors, while biochemical findings should be interpreted according to the completeness of the available phosphate-metabolism assessment.

## 4 Conclusion

Phosphaturic mesenchymal tumor is an important but rare differential diagnosis for jugular foramen masses. In this patient, a tumor arising within the jugular bulb was associated with venous outflow occlusion and mild abnormalities of phosphate metabolism without symptomatic osteomalacia. Gross-total resection through a Fisch type A infratemporal fossa approach was followed by normalization of phosphate-related biochemical indices and a favorable short-term neurological outcome. Recognition of subtle phosphate abnormalities together with careful radiological, pathological, and skull-base surgical assessment may facilitate timely diagnosis and treatment.

## Data Availability

The original contributions presented in the study are included in the article/Supplementary Material, further inquiries can be directed to the corresponding author.
